# Improving knowledge and behaviours related to the cause, transmission and prevention of Tuberculosis and early case detection: a descriptive study of community led Tuberculosis program in Flores, Indonesia

**DOI:** 10.1186/s12889-016-3448-4

**Published:** 2016-08-08

**Authors:** Christa Dewi, Lesley Barclay, Megan Passey, Shawn Wilson

**Affiliations:** University Centre for Rural Health, School of Public Health, University of Sydney, Lismore, NSW Australia

**Keywords:** Tuberculosis, Knowledge, Behaviours, Early case finding, Community-based, Qualitative research, Indonesia

## Abstract

**Background:**

The community’s awareness of Tuberculosis (TB) and delays in health care seeking remain important issues in Indonesia despite the extensive efforts of community-based TB programs delivered by a non-government organisation (NGO). This study explored the knowledge and behaviours in relation to TB and early diagnosis before and after an asset-based intervention designed to improve these issues.

**Methods:**

Six villages in Flores, Indonesia were purposively selected to participate in this study. Three villages served as intervention villages and the other three villages provided a comparison group. Data collection included interviews, group discussions, observations, field notes and audit of records.

**Results:**

In total, 50 participants across six villages were interviewed and three group discussions were conducted in the intervention villages supplemented by 1 – 5 h of observation during monthly visits. Overall, participants in all villages had limited knowledge regarding the cause and transmission of TB before the intervention. The delay in health seeking behaviour was mainly influenced by ignorance of TB symptoms. Health care providers also contributed to delayed diagnosis by ignoring the symptoms of TB suspects at the first visit and failing to examine TB suspects with sputum tests. Stigmatisation of TB patients by the community was reported, although this did not seem to be common. Early case detection was less than 50 % in four of the six villages before the asset-based intervention. Knowledge of TB improved after the intervention in the intervention villages alongside improved education activities. Early case detection also increased in the intervention villages following this intervention. The behaviour changes related to prevention of TB were also obvious in the intervention villages but not the comparison group.

**Conclusion:**

This small project demonstrated that an asset-based intervention can result in positive changes in community’s knowledge and behaviour in relation to TB and early case detection. A continuing education process is like to be required to maintain this outcome and to reach a wider community. Promoting community involvement and local initiatives and engaging health care providers were important elements in the community-based TB program implemented.

**Electronic supplementary material:**

The online version of this article (doi:10.1186/s12889-016-3448-4) contains supplementary material, which is available to authorized users.

## Background

Indonesia was ranked fourth in the world in relation to Tuberculosis (TB) cases in 2010 [1]. Despite the good performance of the National TB Control Program (NTP) in achieving the international targets for case detection (>70 %) and treatment success (>85 %), early diagnosis and treatment, which are considered as important indicators of success in TB control programs, remain the main challenge in Indonesia [2, 3]. There are various factors associated with the delay in the diagnosis and treatment of TB that have been documented from other studies. These include low education level, lack of knowledge about TB, low awareness of TB, non-biomedical health belief systems, and stigma [4].

The TB program in Indonesia is delivered through government and non-governmental organisations (NGOs). Community Development (CD) Bethesda is one of the NGOs in Indonesia that has delivered a community-based TB program in Eastern Indonesia for over 10 years. This program aimed to increase the community’s awareness of TB and to decrease the incidence of TB through education and health promotion, provide training for TB health volunteers, deliver Directly Observed Therapy (DOT) medication to TB patients, provide food supplements during the treatment of TB patients, and to subsidise healthy housing and income generating programs for successfully treated patients (CD Bethesda 2007, 2008, 2009, 2010, 2011, unpublished reports). Despite extensive efforts since 2000 by CD Bethesda and others, CD Bethesda’s biannual reports (CD Bethesda 2007, 2008, 2009, 2010, 2011, unpublished reports) show that people’s awareness of TB and delays in reporting the disease remain major issues in the NGO’s service areas, particularly in Eastern Indonesia.

Health-seeking behaviour and knowledge about and understanding of TB is crucial as this may reduce or increase the transmission of the disease [5]. Given the limitations found with their traditional programs, CD Bethesda decided to evaluate an alternative approach in one of its services areas in Flores, Indonesia. This approach tests a new strategy to engage people in addressing issues related to TB and to improve people’s awareness of TB. The main goal was to see if the new strategy could improve early case finding and prevention activities at the village level.

This paper reports part of a larger study that investigated whether an asset-based approach that emphasises positive capabilities and nurtures the strengths and resources of local community, with stakeholders in a small group of rural Indonesian villages in Sikka District, Flores, Indonesia could improve early identification and prevention of tuberculosis. Another three similar villages provided a comparison group. The asset-based intervention was implemented and evaluated with TB leadership groups in the three intervention villages. The intervention consisted of an initial workshop to shift people’s understanding, followed by a range of other activities. The goal of the initial workshop was for the community to work differently and focus on their strengths rather than their problems or needs, to identify their strengths or assets (knowledge, skills, resources, etc.) and to develop a shared vision and action plan to improve prevention activities and early case finding of TB. Follow-up activities included group meetings, monthly visits, observation, with records kept by the researcher in field notes, and informal discussions. All were conducted as part of the intervention to observe any changes in the villages, provide support to groups or leaders, monitor the groups in implementing their action plans and investigate the consequences of working with an asset-based approach.

The aim of this paper is to explore the knowledge and behaviours related to TB causation, transmission, prevention, and early diagnosis before and after the implementation of the new program into the existing TB program in the three intervention villages and compare the results with three similar comparison villages.

## Methods

### Study setting

Flores is part of the East Nusa Tenggara province. Flores is split into eight regencies (local government districts), which are West Manggarai, Manggarai, East Manggarai, Ngada, Nagekeo, Ende, Sikka, and East Flores.

The study was conducted within CD Bethesda’s service area, which covers 11 villages in Sikka District. The district is made up of 21 sub-districts and 147 villages. This district has the second highest number of TB cases in Flores and ranks 6th in contributing high numbers of TB cases in East Nusa Tenggara in 2012, with 316 new cases of TB (from a population of 300,328 people) and a case detection rate of 48.7 % in 2012 [6].

The district is served by 68 health facilities; one district hospital, two private hospitals, 62 public health centres, and three sub-public health centres. The majority of inhabitants are small-scale farmers growing mainly rice, corn, cassava, cacao, coconut and cashew nuts, and some are fishermen. Sikka district has similar characteristics with other districts in Flores.

### Study population and sampling

Six villages were purposively selected for the study based on identified criteria: willingness to participate in the study; active TB cadres; and low case finding of TB and TB prevention activities.

The first author has been working with CD Bethesda for over 10 years as a planning, monitoring and evaluation officer and routinely visits Flores. The selection of three intervention villages therefore was decided by the researcher’s colleague (who was assigned as an Area Manager of CD Bethesda’s office in Flores) to reduce possible bias. The Area Manager decided which villages would receive the asset-based community led TB program and which three villages would serve as the comparison villages.

The study population consisted of all TB patients, ex-TB patients, village TB volunteers, village leaders, and TB leadership group members in each village. In total 50 people across six villages were recruited for interview using a purposive sampling method based on their status as participants in CD Bethesda’s TB program.

The study was approved by the Human Research Ethics Committee (HREC) of the University of Sydney, Australia and the board of CD Bethesda in Indonesia. Following an explanation of the study and being given a written information sheet, all participants signed the consent form written in Bahasa Indonesian. No incentives were offered to the participants other than meals and drinks during the workshop and group meetings with the TB leadership groups in the three intervention villages.

Tuberculosis leadership groups were only established in the three villages that received the asset-based intervention. The TB leadership groups consisted of representatives of village government, Posyandu cadres (an integrated health service for under five babies or elderly at the village level implemented by voluntary local community (cadre) and public health centre staff), village TB volunteers, TB and ex-TB patients, community members, local leaders and health workers. The TB leadership group members, who attended the groups’ meetings were included in the group discussions. There were between 18 – 30 TB leadership group members who participated in the group discussions in each village.

### Data collection and analysis

Interviews with 17 TB patients, 16 ex-TB patients, 8 village TB volunteers, and 9 village leaders across all six villages were carried out in order to explore the knowledge, behaviours and stigma related to TB and early case detection. These people were relatively equally distributed across each village. Each interview was conducted in the participants’ house or village leaders’ office and took around 30 min to 1 h to complete. The first author, based on her experience and relevant literature including World Health Organization (WHO) guidelines, developed the guide used for the interviews. The interview guide included socio demographic characteristics (sex, age, level of education, occupation) and open-ended questions related to history of the symptoms and health-seeking behaviour, knowledge and perception of TB, and perceived stigmatisation (see Additional file 1).

During the interviews with TB patients, the first author also conducted observations in order to confirm their knowledge and behaviours related to prevention of TB transmission and identify any information found in their house related to TB (poster, leaflet, brochure or booklet about TB). The observations included whether TB patients covered their mouth and nose when coughing or sneezing, where TB patients slept, the condition of their house and bedroom (lighting/sunlight, proper air circulation/ventilation, cleanness, floor, etc.), and whether they have a special container to place their spit. These observations were extended to the village itself.

Group discussions were only conducted in the villages that received the asset-based intervention. Three group discussions with the TB leadership groups in each of the three villages were conducted before the intervention to further explore and confirm the findings of the interviews. The guideline for group discussion included their knowledge of TB symptoms, transmission and prevention of transmission, stigmatisation and why TB patients delay reporting when they have TB symptoms. Further health education was also provided as part of the intervention after information relevant to the study had been collected.

An audit of clinic records was conducted in all six villages to determine whether there was an increase in the number of people who presented for sputum tests following the intervention.

Observations and field notes were completed during the monthly visits to all six villages in order to observe any changes related to early case finding and prevention activities at village level. The researcher spent approximately 1 – 5 h in each village during each visit for observation and discussion with village TB volunteers or community members or TB leadership group members.

The interviews and group discussions were conducted in the Indonesian language. Since the participants also speak in a different local language, the researcher asked village TB volunteers who were parts of the community to be interpreters. This enabled some senior residents, although they understood and spoke Indonesian language, to be able to speak in their own language during the interviews or the group discussions. During interviews and discussions the researcher took notes and made audio recordings with permission from the participants. These were transcribed and allocated to broad categories based on the interview questions. Since the first author was working across three languages, it was not possible to undertake ‘deep’ analysis of meaning, so themes were derived from the questions and answers themselves. Simple descriptive analysis using frequencies was carried out to describe each theme. The analysis was carried out in the Indonesia language and English translation was conducted in the process of report writing.

The interviews and group discussions in the three villages that received the asset-based intervention were repeated six months after implementation of the intervention. The same questions were asked, in order to determine whether there were any changes in responses after the intervention. The baseline interview was also repeated six months later with the same questions and participants in the comparison villages to assess any potential ‘Hawthorne’ effect [7] of the regular visit of the researcher to the villages.

## Results

### Socio-demographic characteristics

Of the 50 individuals interviewed across the villages, 24 participants were from intervention villages and 26 from comparison villages and had similar characteristics (see Table 1 for participant characteristics). Overall, there were more female than male participants (31/50 and 19/50). More than half the participants were TB patients and ex-TB patients (33/50) and the rest were village TB volunteers and village leaders. Half were aged between 35 – 54 years (25/50), and had completed junior high school or higher education (25/50), while 11 participants did not complete elementary school.

**Table 1:** Characteristics of interviewed participants in six villages in Sikka District, Flores

Characteristics	Number of participants		
	Intervention villages (*n* = 24)	Comparison villages (*n* = 26)	Total
Sex			
Male	12	7	19
Female	12	19	31
Age			
15–34	3	3	6
35–54	15	10	25
55 +	6	13	19
Representative			
TB patients	9	8	17
Ex-TB patients	7	9	16
Village TB volunteers	3	5	8
Village leaders	5	4	9
Education			
Not completed elementary school	3	8	11
Elementary school	5	9	14
Junior high school	8	2	10
Senior high school	6	4	10
Vocational college	0	1	1
University	2	2	4
Occupation			
Farmer	10	8	18
Village administration office	6	7	13
Other	4	8	12
Dependant	4	3	7

### Knowledge about TB

#### Source of information on TB

At the beginning of the project, the majority of participants across all six villages said they had heard about TB previously (38/50) and nearly half of these participants (16/38) had information about TB from village TB volunteers during Posyandu activity. Other sources of information included family or friends (8/38), NGOs’ staff (8/38), health workers (5/38), and radio (1/38). No posters/brochures or banners related to TB were found in any villages.

### Causes of TB

Before the project, when the participants across all six villages were asked the causes of TB, only two people mentioned ‘a germ’ as its cause and most participants identified non-infectious causes. A range of behavioural, environmental, socio-economic and other causes were identified (see Table 2). Smoking cigarettes (26/50), drinking local ‘brew’ (25/50), and overwork (25/50) were the most commonly mentioned causes of TB.

**Table 2:** Spontaneous responses to questions about knowledge of TB

Knowledge	Number of participants			
	Intervention villages (*n* = 24)		Comparison villages (*n* = 26)	
	Before study	After study	Before study	After study
Causes of TB^a^				
*Infectious cause:*				
Germ	2	17	0	2
Infected by other	7	11	2	2
*Unhealthy habits:*				
Smoking	12	2	14	15
Drinking local brew	10	3	15	13
Overwork	12	3	13	17
Eating betel nut	3	0	3	4
Drinking coffee	3	0	4	2
Lack of good nutrition	6	1	5	3
Lack of rest	3	0	3	0
*Environmental factor:*				
Unclean environment	4	3	4	5
*Socio-economic factor:*				
Poor	1	1	2	1
Stress	2	0	3	4
*Others:*				
Hereditary	3	0	3	3
Black magic	1	0	1	1
Do not get vaccination	0	2	2	0
Do not know	1	0	2	1
Modes of TB Transmission^a^				
*Correct responses*				
Patient coughing/sneezing/talk face to face to others	7	18	2	1
TB patients spitting anywhere	10	13	12	12
Sleeping in the same room with TB patients	6	10	8	9
*Incorrect responses*				
Sharing eating utensils with TB patients	17	5	16	18
Sharing of food/drinking/cigarette	4	3	4	5
Sexual intercourse	1	0	0	1
Patient carrying children	5	1	6	5
Contaminated water	1	0	2	2
Faeces	0	0	2	2
Do not know	2	0	1	0
TB Symptoms^a^				
*Correct response*				
Cough that lasts longer than 2 weeks	3	3	2	0
Persistent cough	14	20	17	18
Coughing up blood	17	19	11	14
Weight loss	16	21	9	10
Sweating during the night	3	19	6	6
Chest pain	8	19	15	13
Loss of appetite	6	13	5	6
Fever	6	15	1	0
Difficult breathing/chest tightening	8	17	9	11
Body malaise	3	9	3	1
Heat in the chest	0	0	2	4
*Incorrect responses*				
Reduced visibility/hearing	3	0	0	0
Sore throat	0	0	2	4
Dry cough	1	0	2	0
Emotional	0	0	1	2
Difficult to sleep	1	0	1	2
Headache	1	0	3	3
Do not know	1	0	1	1

^a^Most people gave more than one answer, so the table reflects this

*“People could get TB if too much working and eating betel nut.. This is for women.. For men, because of drinking local brew and smoking…”* (ex-TB patient, comparison village)

*“My deceased father also got TB. He always coughs up blood and I usually cleaned his blood. Maybe because of it, I also got TB…” *(TB patient, intervention village)

“*I did not think that I could get TB… as I knew TB was inherited… Usually if the parents got TB, their children would also get TB. I don’t have any family who got TB…”* (ex-TB patient, intervention village)

*“TB was a very bad disease.. It was because of black magic, so people did not talk about it…” *(ex-TB patient, intervention village)

After the implementation of the asset-based intervention, although there were still some participants in the intervention villages who indicated unhealthy habits (smoking, drinking, overwork, and lack of good nutrition) and unclean environment, as the basis of TB, these were fewer than before the intervention (see Table 2). No participants indicated that TB was inherited or due to black magic, lack of rest, eating betel nut and drinking coffee as the causes of TB. Most participants identified ‘a germ’ (17/24) as the cause of TB. Being infected by others (11/24) and no TB vaccination (2/24) were also mentioned as causes of TB. In the comparison villages however, there was little change in perception of the causes of TB (see Table 2).

### Modes of TB transmission

Before the project, less than half the participants across all six villages indicated coughing or sneezing or talking face to face with TB patients (9/50), spitting anywhere (22/50), and sleeping in the same room with a TB patient (14/50) as sources of transmission. Most of the participants identified incorrect sources of TB transmission, such as sharing of eating utensils with TB patients, carrying children, sharing of food or cigarettes or drinking with TB patient, contaminated water, faeces and sexual intercourse (see Table 2).

“*We cannot share eating utensils and sleep in the same room.. If I slept with my children, I had to sleep with my back to them.. My children cannot eat/drink my leftover meal/drink. I also spitted in a covered can filled with ash and covered my mouth when I talk to others to prevent the transmission to other people…*” (ex-TB patient, intervention village)

After the implementation of the asset-based intervention, although there were still participants who indicated sharing of eating utensils or food/drinking/cigarette with a TB patient and carrying children as the modes of TB transmission, most of the participant in the intervention villages identified coughing or sneezing or talking face to face with TB patients as the commonest mode of TB transmission (see Table 2). No participant indicated sexual intercourse and contaminated water as the modes of TB transmission. In the comparison village, on the other hand, after the study, there was little change (see Table 2).

### TB symptoms

At the beginning of the project, persistent cough (31/50), coughing of blood (28/50), weight loss (25/50) and chest pain (23/50) were the most common symptoms mentioned by participants both in the intervention villages and comparison villages. Other possible symptoms mentioned included difficulty breathing/chest tightening, loss of appetite, sweating during the night, chest pain, fever, and body malaise. Only a few participants indicated incorrect TB symptoms (see Table 2). During discussions with TB leadership groups in the three intervention villages, cough, weight loss and weakness were mentioned as the most obvious indicators of TB.

*“If we see a person has got prolonged cough and he/she is getting skinny and skinny, even cannot do any work anymore, we suspect he/she might have TB…” *(TB leadership group member, intervention village)

After the implementation of the asset-based intervention, of the 24 participants in the intervention villages, there were more participants that mentioned weight loss (from 16 to 21 people), persistent cough (from 14 to 20 people), coughing up of blood (from 17 to 19 people), chest pain (from 8 to 19 people) and sweating during the night (from 3 to 19 people) as the most common symptoms of TB. In the comparison villages however, although the number of participants who identified correct TB symptoms increased, these numbers were less than those in the intervention villages (see Table 2).

### Activities undertaken to improve knowledge

There were no program activities conducted in any of the six villages in relation to increasing community’s knowledge about TB during the six months period before the study. This was only done through personal approaches from village TB volunteers to TB suspects and their family.

**Fig. 1:**
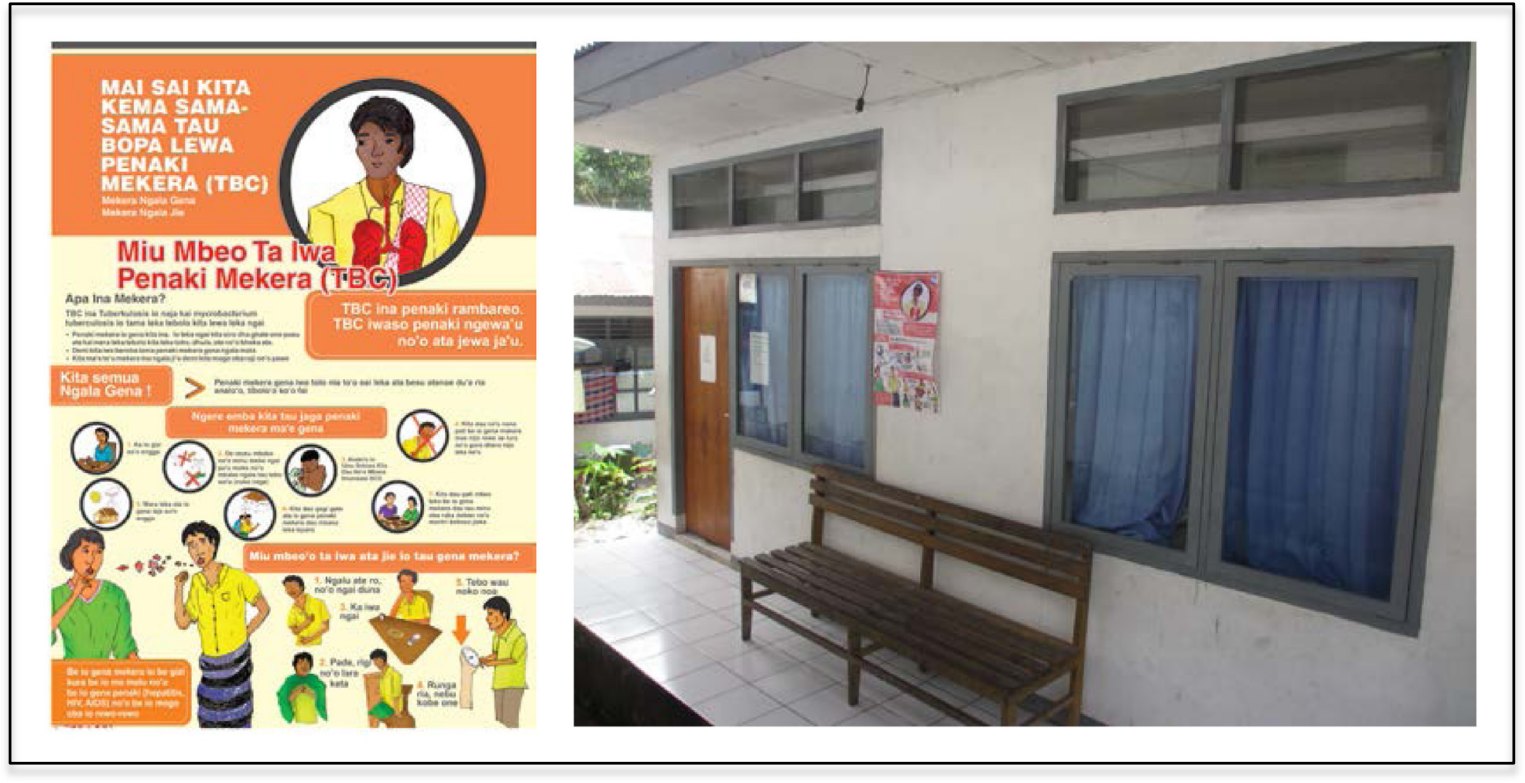
Poster about TB which was designed by a TB leadership group member

After the implementation of the asset-based intervention, the activities to increase community’s knowledge related to TB increased in the intervention villages. This occurred in a variety of ways; for example through Posyandu for under-five children or elderly (2 – 3 times in each village), community meetings (1 – 3 times in each village), as well as through posters which were designed by a TB leadership group member from one village and which used local languages. The poster included information about what TB is, its symptoms and transmission, and how to prevent getting TB. The posters were displayed in public areas, such as the Posyandu, the village administration office, and the public health centre (see Fig. 1). For community members, including TB patients and ex-TB patients, the posters were designed in the form of a calendar.

In the comparison villages, on the other hand, only one village delivered education about TB through Posyandu for under-five children during the period of this study.

### Stigma

Most participants across the six villages (39/50) indicated that TB patients were not stigmatised. Others thought people were friendly but avoided TB patients (4/50). Only six participants (four from intervention villages and two from comparison villages) mentioned that people avoided TB patients.

“*My friends stay away from me because they are afraid to get infected…*” (TB patient, intervention village)

Indeed, five of the TB patients and ex-TB patients (all were from intervention villages) reported that they hid their disease from their neighbours and friends because they were ashamed and afraid of being avoided. This was also confirmed during group discussion in three villages.

“*I did not tell my friends because I am afraid they will mock me. They will tell my other friends not to come close to me so they will not get infected. I felt uncomfortable…*” (ex-TB patient, intervention village)

*“People will be ashamed if they get TB because they are afraid that other people will label them as a TB offspring and then avoid them… Actually people do not avoid them or discriminate against them, but they prefer to hide their disease…”* (TB leadership group member, intervention village)

When the participants across all villages were asked their reaction when/if diagnosed with TB, of 50 participants, 16 people said they feared or would fear death, eight people felt or would feel sad, seven people felt or would feel ashamed, and three people felt or would feel fear of being avoided. Only two participants said that they felt relieved when they knew they had TB because they knew they could get treatment immediately.

“*I was so scared and I just felt I would surely die… What I heard, TB cannot be cured, so if someone got TB, he/she will die…*” (ex-TB patient, intervention village)

### Health-seeking behaviour

All the TB patients and ex-TB patients (*n* = 33) across the six villages were asked how long they had the symptoms before they sought treatment. Health seeking behaviour was generally delayed until the symptoms were getting severe or interfered with their work or other activities. Only six participants reported they had experienced a cough of only two or three weeks duration before seeking treatment. About one third of participants had TB symptoms for about one month to five months. However, nearly half of participants reported that they had a cough that lasted for three months to one year (15/33) and six participants reported they had a cough for more than one year. More than half of participants (20/33) said they did not know or had never thought that their symptoms were due to TB. During discussions with TB leadership groups in the three intervention villages, lack of knowledge and awareness of TB symptoms, fear of being avoided, perception that TB cannot be cured, and feeling ashamed were mentioned as the common reasons why people delay reporting the symptoms.

“*I had been sick for two years. I went to a traditional healer but he suggested to me to go to the hospital. Before that, my friend who also had got TB suggested to me to check my sputum because he thought I might have got TB as well. I insisted that it was not TB because I have never lived with TB patients… I have heard about TB, but I did not think I would get TB. I thought I have got AIDS instead of TB because I was a driver; I often drove overseas tourists, ate together with them, even I also wore their swim pant…”* (TB patient, intervention village)

*“I was sick for more than one year and getting skinny and skinny. I took my own medicine, I went to a traditional healer, I went to the hospital and prayer, but I was still sick. I did not think I got TB because I did not know about TB previously.... TB cadre suggested to me to check my sputum at the public health centre…”* (ex-TB patient, comparison village)

“*I thought I will not get TB because I do not smoke nor drink liquor…*” (ex-TB patient, comparison village)

Most of the TB patients and ex-TB patients sought treatment. Some went to the public health centre after their TB symptoms began (16/33) and the rest of them went to hospital (6/33), applied self- medication using traditional medicine or over the counter drugs (5/33), went to a private practitioner (4/33) and a traditional healer (2/33). Six of them reported that they were not examined for TB the first time they went to a public health centre and were only given cough medicine. Meanwhile, four of them said they were examined by sputum test, but the results were negative and then they were asked to do an X-ray test.

“*I went to the public health centre and was given cough medicine… I had high fever for more than one month and my family brought me to the hospital… I was examined by X-ray and then was referred back to the public health centre to do a sputum test …*” (ex-TB patient, intervention village)

“*I was coughing up blood… I did a sputum check, but the result was negative. Then I was examined by X-ray and the result was positive*” (ex-TB patient, comparison village)

### Early case finding

The District Health Office set the target for case finding and positive tests for each village based on the population size. According to the target of NTP, each village was expected to achieve >70 % of the target for case finding.

Before the project, record audit data showed the number of people who presented for sputum tests across the six villages varied between 3 to 74 people in each village (see Table 3). It was equally variable across intervention and comparison villages. Although the target for case finding set by the District Health Office was not achieved 100 % in any of the intervention villages, one (village C) achieved the target for case finding (>70 %) before the intervention. The case finding in one comparison village (village F) even extended beyond the target and was about 2.5 times for the target. This was because the village TB volunteer also reached people from her neighbour village.

“*I also make a living from selling traditional medicine. I usually walk, not only within this village, but also the neighbour village, to sell my medicine. So, while I walk to sell my medicine, I also try to find TB suspects. If I know someone has TB symptoms, I will ask them regarding the symptoms and then give them a pot for the sputum, then I will bring the pot to the public health centre…*” (village TB volunteer, comparison village)

After the implementation of the asset-based intervention, the number of people who presented for sputum tests in all three of intervention villages increased and all villages achieved the target (>70 %). In one of intervention villages, this number increased dramatically (from 3 to 25 people). In the comparison villages, on the other hand, although the number of TB suspects who presented for sputum tests in one of the comparison villages increased after the study (from 22 to 33 people), this did not achieve the government set target. In the two other comparison villages, the number of sputum tests actually decreased compared to before the study. See Table 3.

In relation to the number of positive tests, three of the six villages (one intervention village and two comparison villages) achieved the target of positive tests before the project (see Table 3). After the implementation of the asset-based intervention, one intervention village consistently achieved the target of positive tests; even increasing this compared to before the program (from 1.25 to 1.5 times of the target). In the other two villages, although they did not achieve the target, the number of positive tests also increased. In the comparison villages however, the number of positive tests decreased in all villages and all did not achieve the target.

**Table 3:** The number of people presenting for sputum testing 6 months before and after the study

Village	Total population (*in 2013*)	Target of case finding^a^	Target of positive tests^b^	Number of people presenting for tests		Number of positive tests	
				Before study	After study	Before study	After study
Intervention villages							
Village A	1952	41	4	20	29	5	6
Village B	821	20	2	3	25	0	1
Village C	2536	53	5	45	60	1	3
Comparison villages							
Village D	1877	40	4	24	7	4	0
Village E	2526	53	5	22	33	3	1
Village F	1327	28	3	74	17	4	1

^a^Target for case finding was determined by the District Health Office (210/100000 X total population)

^b^Target for positive tests was determined by the District Health Office (1:10 of number of case finding)

### Prevention activities and behaviour changes

Before the project, when asked how to prevent TB, more than half of participants indicated environmental hygiene (29/50) and good nutrition (27/50) were the most important factors. This finding was also confirmed during the workshop with TB leadership groups in the three intervention villages. During the workshop, when participants were asked to list factors that contribute positively and negatively in relation to TB prevention, they also identified good nutrition, healthy and clean behaviours, and environmental hygiene as the important factors. These factors were taken into consideration when TB leadership groups developed their one-year action plans to improve prevention of TB that were part of the intervention.

**Fig. 2:**
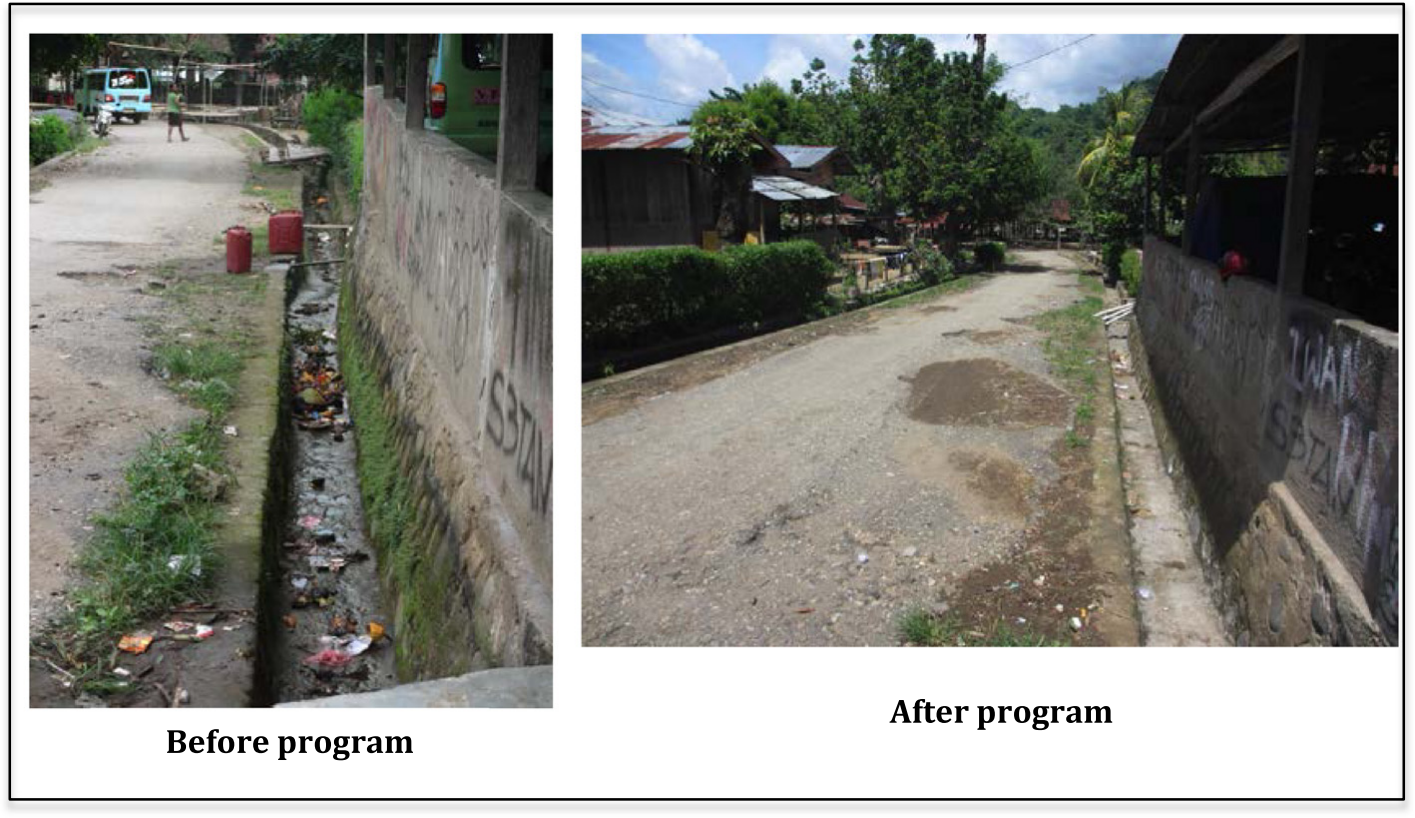
The same streetscape showing behaviour change in relation to environmental hygiene

**Fig. 3:**
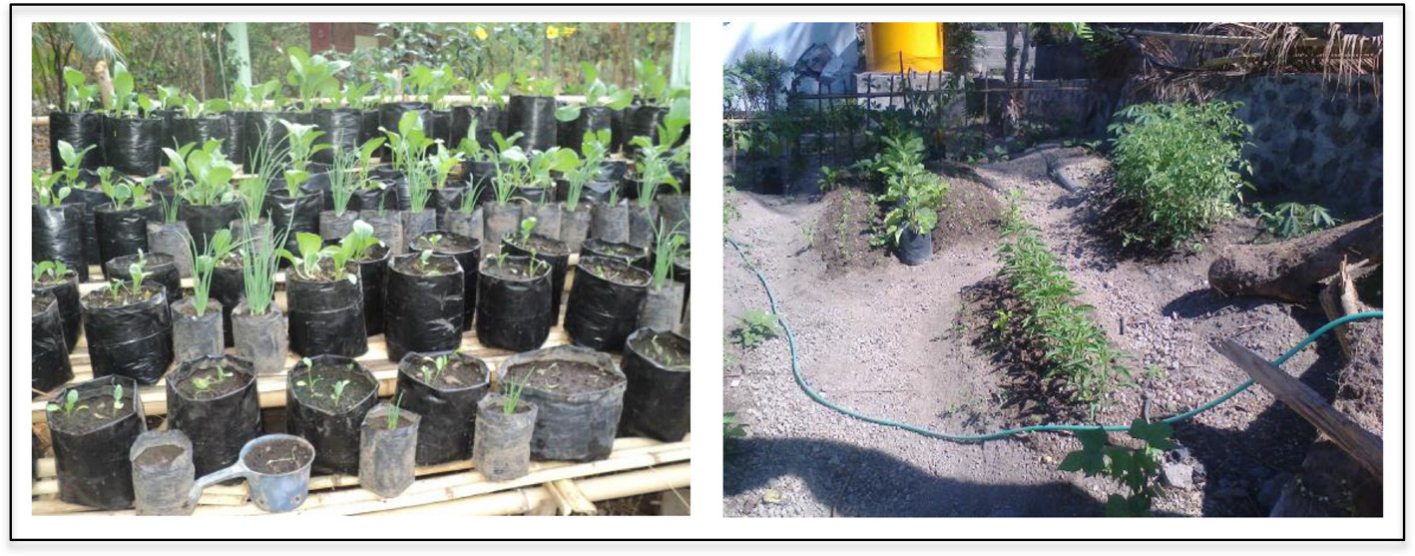
The TB leadership groups’ member planting vegetables in their yard

At both household and village level, behaviour changes related to prevention activities for TB were obvious in the intervention villages after the implementation of the asset-based intervention. Previously, people closed their windows during the day. After the intervention, people started opening their windows during the day. And every Friday, the community cleaned public areas and the street in front of their houses (see Fig. 2). Planting some vegetables in polybags or in their yard to improve family nutrition also occurred after the intervention (see Fig. 3). In one village, the villagers initiated a nutrition post to improve the nutrition of malnourished children because they recognised that children are very vulnerable to getting disease if they are malnourished. People were also more openly talking about TB. Indeed, some people reported to the village TB volunteers when their family members had TB symptoms and asked the village TB volunteers to bring them to the public health centre. This had not occurred previously.

In the comparison villages, on the other hand, there was no obvious change in relation to prevention activities towards TB. In one comparison village however, the village TB volunteers became more enthusiastic in finding TB suspects, and in one other village, a village TB volunteer started to plant some vegetables in her yard.

## Discussion

This study revealed limited knowledge and some misunderstanding about TB among participants across the whole study area. Tuberculosis is caused by a bacterium, but only 2 among 50 participants mentioned ‘a germ’ as a cause of TB. This result accords with findings by Portero et al. [8] in Manila, which reported that only 25 % of those interviewed knew that TB is caused by ‘a germ’. Smoking cigarettes, drinking local ‘brew’ , and working too hard were identified as the commonest perceived causes of TB in this study. These non-infectious causes were also reported in other studies conducted in Tanzania [5], Bali [9], and Jogjakarta [3]. In Manila by Portero et al. [8] reported that nearly one third of those interviewed believed that TB was inherited. Watkins and Plant [9] also reported that people in Bali related causes of TB to being inherited, magic, and an unclean environment. Similar understandings were also found in this study. It was thought TB might be transmitted through sharing of eating utensils and food/drinking/cigarettes, carrying children, and sexual intercourse. Again, similar findings regarding beliefs on how TB was transmitted through physical contacts such as sharing of cups, having sexual intercourse with a TB patient, and from mother to children have been reported in a study in Zambia [10]. Despite the misconceptions around cause and modes of transmission of TB, participants in this study had some knowledge that TB is a transmissible disease and might be transmitted by either direct or indirect contact. More than half of the participants had obtained information about TB from the village TB volunteers, family or friends, NGO staff, and health workers. However, such information was likely to be misinformed or misunderstood. There is a need to strengthen health education that stresses the correct cause and mode of transmission of TB.

Persistent cough, coughing up blood, weight loss and chest pain were the main symptoms associated with TB among the participants in this study. This implies that the participants had good knowledge regarding the clinical manifestations of TB. However, nearly half of the TB patients and ex-TB patients interviewed in this study had symptoms for between 3 months to one year before they sought health care. Once the symptoms became severe or interfered with their work or activities, they went to health facilities. Most of the participants were unaware that their symptoms were associated with TB until they were diagnosed. This is consistent with a study in Bali [9] which reported that delayed health seeking behaviour was attributed to poor awareness of the seriousness of TB symptoms, stigma and accessibility of treatment services. An Ethiopian study also reported that about 45 % of TB suspects had a cough that lasted for 3 months to one year before they took health care action because they did not consider their symptoms to be severe [11].

Nearly half of the TB patients and ex-TB patients interviewed in this study went to the public health centre for their symptoms, but most of them were not examined or failed to be examined with a sputum test. This implies that health care providers also contributed to delayed diagnosis that could increase the risk of death and TB transmission in the community. A similar finding from Jogjakarta reported the contribution of health care providers in delaying diagnosis by frequently failing to examine TB suspects with smear microscopy [3].

The results of this study indicated that most TB patients did not feel stigmatised by the community. About 30 % of TB patients and ex-TB patients interviewed in this study reported their friends or neighbours avoided them when they knew the patient had TB. A minority of them (less than 30 %) also hid their disease because they were ashamed and afraid of being avoided. Therefore it appears that stigma was not a major contributor to people delaying seeking treatment.

In the intervention villages, the participants’ knowledge regarding the cause of TB, modes of transmission and TB symptoms improved after the implementation of the intervention, as anticipated. Although there were still misconceptions about the cause of TB and its transmission, this study showed that most of the participants had better knowledge compared to the villages that did not receive the intervention. The intervention also improved TB leadership groups’ behaviours related to prevention of TB through the improvement of environmental hygiene, nutrition for families and under-five children and their own educational activities. This might be because the health education activities and interaction among TB leadership groups’ members improved during the implementation of the asset-based intervention and facilitated peer support for these activities.

The asset-based intervention not only resulted in improving the knowledge and awareness of TB and prevention of TB, but also in improving early case detection in the intervention villages. This study found that case finding in all the intervention villages increased after the intervention, with two of them achieving the target in case finding. In the comparison villages, on the other hand, the target for the number of people presenting for a TB test was not achieved. This suggests that the improvement of knowledge of TB, improved awareness of TB, and this in turn improved early case detection. As Lambert and Stuyft stated, health education regarding TB aims to reduce delay in health care seeking and increase case detection [12].

Kretzmann and McKnight [13] suggest that community-based interventions are only successful if the local community commit to investing their skills and resources in the program. Therefore, it is important to start a community-based intervention by discovering and mobilising what is present in the community, such as the capacities and assets of local individuals, associations, and institutions. The WHO [14] also recommended that the approach to community-based TB programs should include people who are directly affected by TB and community members in planning, implementing and evaluating interventions from the very beginning. The results of this study showed that the asset-based intervention that emphasises and values the capacity, skills and knowledge of local communities improved the leadership capacity of TB leadership groups in the intervention villages in managing their own TB program that in turn resulted in improved TB care outcomes. This included working differently to improve early case detection and prevention of TB by taking control of their own TB program instead of depending on a TB program determined by the NGO.

There were a number of limitations in this study. This included, first, the small number of participants involved in this study and that the samples may not have been representatives. The selection of the sample was pragmatic not random as the intervention was conducted for 8 months involving monthly visits to 6 villages making it difficult to involve a larger sample. Secondly, it is difficult to accurately measure the effectiveness of this intervention in relation to the improvement of communities’ knowledge and awareness of TB and early case detection. However, the strengths were this study was clearly community-based and this intervention has encouraged community involvement and strengthened local initiatives to improve their health.

## Conclusion

This study showed that most of the participants had limited knowledge and misunderstood causes and transmission of TB before the asset-based intervention occurred despite a decade of conventionally focussed TB programs. This lack of knowledge of the symptoms of TB appeared to influence health seeking behaviour. This small study however has shown that an asset-based intervention working with local leaders resulted in positive changes in relation to knowledge and awareness of TB, the community’s behaviour as well as early case detection. A continued mentoring and support education process is required to improve the knowledge and awareness of a wider community and to maintain the behaviour changes. Engaging health care providers in this process is also important so they can improve early diagnosis.

## Abbreviations

CD, community development; DOT, Directly Observed Therapy; NGO, non-government organisation; NTP, National TB Control Program; TB, tuberculosis; WHO: World Health Organization

## Additional file

Additional file 1: Interview guide. (DOCX 81 kb)
