# Supplementary material for: Improving knowledge and behaviours related to the cause, transmission and prevention of Tuberculosis and early case detection: a descriptive study of community led Tuberculosis program in Flores, Indonesia
Source: BMC Public Health. 2016 Aug 8;16:740. doi: 10.1186/s12889-016-3448-4 (PMC4977733; doi:10.1186/s12889-016-3448-4)
Supplement: Additional file 1: — Interview guide. (DOCX 81 kb) [file 12889_2016_3448_MOESM1_ESM.docx]

Additional file 1: Interview guide

**Table 1. Interview guide**

| ***Interview guide***  *The following questions were used for guidance of participant interviews.*  *Knowledge*   - Have you heard about TB before? How did you hear about TB? - What are the signs and symptoms of TB? - How can a person get TB? (Causes and transmission) - How can a person prevent or minimise getting TB? - In your opinion, who can be infected with TB?   *Attitude & behaviours* (modified as appropriate depending on TB experiences)   - Do/did you think you could get TB? - What the first thing you do/did, when you thought you had symptoms of TB? - If you had symptoms of TB, at what point would you go to the health facility? - When you have/had symptoms of TB, how long did you wait before seeking treatment? - What would/was your reaction, when you found out that you have TB? - Who would/did you talk to about your illness if you had TB? - What did/do you do to minimise the transmission of TB to your family members and others? - How is a TB patient regarded/treated in your community?   *TB awareness and source of information*   - Do you feel well informed about TB? - Do you want more information about TB? - What are the sources of information that you think can most effectively reach people like you with information on TB? - What worries you the most, when you think about TB? |
| --- |
